# A Multicenter Study To Evaluate Ceftaroline Breakpoints: Performance in an Area with High Prevalence of Methicillin-Resistant Staphylococcus aureus Sequence Type 5 Lineage

**DOI:** 10.1128/JCM.00798-19

**Published:** 2019-08-26

**Authors:** Ayesha Khan, Lina M. Rivas, Maria Spencer, Rodrigo Martinez, Marusella Lam, Pamela Rojas, Lorena Porte, Francisco Silva, Stephanie Braun, Francisca Valdivieso, Margareta Mv̈lhauser, Mónica Lafourcade, William R. Miller, Patricia García, Cesar A. Arias, Jose M. Munita

**Affiliations:** a Center for Antimicrobial Resistance and Microbial Genomics and Division of Infectious Diseases, University of Texas Health Science Center, McGovern Medical School, Houston, Texas, USA; b Department of Microbiology and Molecular Genetics, University of Texas Health Science Center, McGovern Medical School, Houston, Texas, USA; c MD Anderson Cancer Center, UT Health Graduate School of Biomedical Sciences, Houston, Texas, USA; d Genomics and Resistant Microbes Group, Facultad de Medicina-Clinica Alemana, Universidad del Desarrollo, Santiago, Chile; e Millennium Initiative for Collaborative Research on Bacterial Resistance (MICROB-R), Millennium Science Initiative, Santiago, Chile; f Departamento de Laboratorios Clínicos, Escuela de Medicina, Pontificia Universidad Católica de Chile, Santiago, Chile; g Hospital Padre Hurtado, Santiago, Chile; h Hospital Clínico Universidad de Chile, Santiago, Chile; i Hospital Militar, Santiago, Chile; j Hospital Dr. Luis Calvo Mackenna, Santiago, Chile; k Hospital Dipreca, Santiago, Chile; l Clínica Santa María, Santiago, Chile; m Molecular Genetics and Antimicrobial Resistance Unit, International Center for Microbial Genomics, Universidad El Bosque, Bogotá, Colombia; n Center for Infectious Diseases, University of Texas Health Science Center, School of Public Health, Houston, Texas, USA; Mayo Clinic

**Keywords:** ceftaroline, disk diffusion, Etest, MIC, MRSA, breakpoints

## Abstract

Ceftaroline (CPT) is a broad-spectrum agent with potent activity against methicillin-resistant Staphylococcus aureus (MRSA). The sequence type 5 (ST5) Chilean-Cordobés clone, associated with CPT nonsusceptibility, is dominant in Chile, a region with high rates of MRSA infections. Here, we assessed the *in vitro* activity of CPT against a collection of MRSA isolates collected between 1999 and 2018 from nine hospitals (*n* = 320) and community settings (*n* = 41) in Santiago, Chile, and evaluated performance across testing methodologies. We found that our hospital-associated isolates exhibited higher CPT MIC distributions (MIC_50_ and MIC_90_ of 2 mg/liter) than the community isolates (MIC_50_ and MIC_90_ of 0.5 mg/liter), a finding that was consistent across time and independent of the culture source. High proportions (64%) of isolates were CPT nonsusceptible despite the absence of CPT use in Chile. Across methodologies, the Etest underestimated the MIC relative to the gold standard broth microdilution (BMD) test (MIC_50_ and MIC_90_ of 1 and 1.5 mg/liter, respectively). There was low (∼51%) categorical agreement (CA) between Etest and BMD results across CLSI and EUCAST breakpoints. The recent revision of CLSI guidelines abolished “very major error” (VME) from the previous guidelines (81%), which perform similarly to the EUCAST guidelines. The level of concordance between CLSI and EUCAST for BMD testing and Etest was >95%. Disk diffusion performed poorly relative to BMD under CLSI (CA, 55%) and EUCAST (CA, 36%) guidelines. Comparison of EUCAST to CLSI for disk diffusion (with EUCAST used as the reference) showed low agreement (CA, 25%; VME, 70%). In summary, CPT-nonsusceptible MRSA are dominant in clinical settings in Chile. Our results provide data to support the reevaluation of CPT breakpoints and to improve agreement across methodologies and agencies.

## Abstract (precis)

Ceftaroline (CPT) is a broad-spectrum agent with potent activity against methicillin-resistant Staphylococcus aureus (MRSA). The sequence type 5 (ST5) Chilean-Cordobés clone, associated with CPT nonsusceptibility, is dominant in Chile, a region with high rates of MRSA infections.

## INTRODUCTION

Staphylococcus aureus is a major human pathogen with the ability to evolve and adapt under conditions of antibiotic selective pressure, making methicillin-resistant S. aureus (MRSA) particularly problematic due to resistance to commonly used antibiotics (1). MRSA is a leading cause of health care-associated infections (2) and has also been identified as an important pathogen in community settings (community-associated MRSA [CA-MRSA]), causing epidemics in several locations around the world (3, 4). Dissemination of MRSA largely occurs through the activity of genetic lineages that expand into and dominate specific geographic areas (4). For instance, the rapid dissemination of the sequence type 8 (ST8) USA300 clone established it as an endemic CA-MRSA lineage in North America (5), while a closely related genetic lineage (designated USA300-LV) dominates in some areas of South America (6, 7). USA300-LV is prevalent in Colombia, Venezuela, and Ecuador (3), while different representatives of clonal complex 5 (CC5) predominate in Brazil, Chile, and Peru, indicating extensive regional diversity in MRSA population dynamics (8). In Chile and Peru, the prevalence of MRSA is high. Indeed, in a recent prospective study of S. aureus bacteremia using samples from three hospitals in Chile, 45% of all bloodstream isolates were MRSA, and the majority (∼90%) belonged to the ST5 Chilean-Cordobés lineage, previously reported to be widely disseminated across the region (8–11).

Resistance to antistaphylococcal penicillins and most other β-lactams in S. aureus is mainly attributed to *mecA*-encoded low-affinity penicillin binding protein 2a (PBP2a), which is able to perform transpeptidation in the presence of virtually all β-lactam antibiotics (12). Ceftaroline (CPT) is a recently introduced broad-spectrum cephalosporin with remarkable anti-MRSA activity due to its distinct ability to inhibit PBP2a (13). CPT has been approved by the U.S. Food and Drug Administration for the treatment of complicated skin and soft tissue infections (including infections by MRSA) and community-acquired pneumonia (methicillin-susceptible isolates only) and has seen increasing off-label use in treatments of other MRSA infections (14, 15). Of note, low-level CPT resistance in MRSA has been reported around the world, predating the introduction of the drug into hospitals (16–18). On the other hand, high-level CPT resistance remains infrequent and has been attributed to specific substitutions in the penicillin-binding domain of PBP2a (19, 20).

The European Committee on Antimicrobial Susceptibility Testing (EUCAST) has established an MIC of ≤1 mg/liter as the clinical breakpoint for CPT susceptibility. However, EUCAST’s definitions of resistance differ between isolates obtained from pneumonia (>1 mg/liter) and those obtained from other clinical sources (>2 mg/liter). Isolates exhibiting CPT MICs of 1 mg/liter fall in a category denominated “area of technical uncertainty” (ATU) (21). The Clinical and Laboratory Standards Institute (CLSI) recently refined its recommendation for CPT breakpoints. In line with EUCAST, isolates with an MIC of ≤1 mg/liter are considered susceptible; however, CPT resistance was established with an MIC of ≥8 mg/liter (the previous breakpoint was ≥4 mg/liter). Isolates exhibiting CPT MICs of 2 to 4 mg/liter fall into the susceptible dose-dependent (SDD) category (22), in contrast to previous CLSI recommendations that placed isolates with a CPT MIC of 2 mg/liter in an intermediate category. In addition, there are major differences between these agencies in terms of disk diffusion techniques and breakpoints. CLSI disk diffusion recommendations are based on a disk with 30 μg of CPT; a diameter of ≤19 mm is defined as representative of resistance, zones 20 to 24 mm in diameter fall into the SDD category, and a zone of ≥25 mm in diameter is considered to represent susceptibility (22). In contrast, EUCAST uses a 5-μg CPT, disk with nonpneumonia isolates defined as resistant if the zone diameter is <17 mm and susceptible if ≥20 mm. Pneumonia-derived isolates are considered resistant or susceptible with a diameter of <20 or ≥20 mm, respectively (21). Both nonpneumonia and pneumonia-derived isolates exhibiting a diameter of 19 to 20 mm fall in the ATU. All breakpoints are listed in Table S1 in the supplemental material.

A recent European surveillance study compared levels of CPT MIC value agreement between Etest and broth microdilution (BMD) and found good reliability for isolates with an MIC of ≤1 mg/liter but not for those with an MIC of ≥2 mg/liter (23). A 2012 international surveillance study reported that 73% (90/123) of MRSA isolates from three Chilean hospitals had high CPT MICs of 2 mg/liter and that all of the isolates belonged to ST5 (18). Similarly, another study from South Korea reported that among all the MRSA isolates in their study that were CPT nonsusceptible (44%, 70/159), all but one belonged to the ST5 lineage and harbored substitutions in PBP2a (24). Thus, previous data suggested that some MRSA lineages, such as ST5, exhibit higher CPT MICs associated with changes in the predicted PBP2a sequence. In this study, we evaluated the *in vitro* activity of CPT against MRSA isolates circulating in Santiago, Chile, using a collection of clinical isolates recovered from various hospitals and the community from 1999 to 2018. Of note, CPT was not available for clinical use in Chile until 2019. In addition, since previous data suggested that CPT susceptibility testing using Etest or disk diffusion is unreliable for isolates with higher MICs (i.e., 1 to 2 mg/liter) (25), we also aimed to evaluate the performance of Etest and disk diffusion methodologies in detecting CPT nonsusceptibility relative to the gold standard BMD and to compare CLSI and EUCAST breakpoints for all methodologies tested.

## MATERIALS AND METHODS

### Bacterial strains.

MRSA clinical isolates were collected between 1999 and 2018 from 9 tertiary-care hospitals in Santiago, Chile. The CA-MRSA isolates were obtained between 2012 and 2017 in a surveillance study of isolates from individuals with no recent history of hospitalizations. All strains were analyzed in a central laboratory. Species identification and the methicillin resistance phenotype were confirmed with matrix-assisted laser desorption ionization–time of flight (MALDI-TOF) and analysis of susceptibility to cefoxitin (disk diffusion), respectively. PCR for *mecA* was performed on all isolates.

### Susceptibility testing.

MICs were determined in triplicate by BMD testing on panels prepared in-house using cation-adjusted Mueller-Hinton (CAMH) broth (Becton, Dickinson, Franklin Lakes, NJ), as recommended by both EUCAST and CLSI guidelines (21, 26, 27). CPT concentrations spanned a doubling dilution range of 0.032 mg/liter to 4 mg/liter. MICs were read after incubation at 35°C in a non-CO_2_ incubator for 16 to 20 h. The Etest (bioMérieux, Marcy l’Etoile, France) was performed on MH agar (Oxoid, Basingstoke, United Kingdom) with incubation at 35°C in the incubator for 16 to 24 h following the manufacturers’ instructions. Disk diffusion testing was carried out in three biological replicates according to EUCAST and CLSI guidelines (27, 28). For EUCAST determinations, disk testing was performed with 5-μg CPT disks (Bio-Rad, Hercules, CA) and CAMH agar (Becton, Dickinson). For CLSI determinations, 30-μg disks (Oxoid) were used. For all susceptibility testing, the same inoculum was prepared for every replicate and the same inoculum was used across testing methodologies performed in parallel.

Quality control testing was performed using S. aureus ATCC 29213 for BMD testing and Etest and S. aureus ATCC 25923 for disk diffusion. If the control did not report an MIC within a range of 0.12 to 0.5 mg/liter, the full experiment was not recorded and repeated. Susceptibilities were analyzed using 2019 EUCAST (21) breakpoints or the newly revised (2019) CLSI criteria. Comparisons were also made using the previous (2018) CLSI criteria (22, 29).

### Data analyses.

Susceptibility results were compared and categorized in relation to BMD testing. Categorical agreement (CA), essential agreement (EA), major errors (ME), very major errors (VME), and minor errors (ME) were evaluated. EA was defined as agreement within ±1 2-fold dilution of the results obtained using the method under evaluation with those obtained by BMD. Etest MIC values were rounded up to the next concentration of the standard doubling dilution scale when necessary. CA was defined as agreement of interpretative results between the method under evaluation and BMD using CLSI or EUCAST, as appropriate. If the MIC value did not stringently fall into the susceptible category or the resistant category, it was classified as representing nonsusceptibility, which included the categories of intermediate susceptibility (CLSI 2018) and SDD (CLSI 2019). Significant differences in MIC values for the hospital-associated MRSA isolates versus the MIC values for the CA-MRSA isolates were assessed with a Mann-Whitney-Wilcoxon *t* test. Variation in the categorization of isolates as susceptible, nonsusceptible (uncategorized, susceptible-dose dependent, and/or intermediate), or resistant across agencies or testing methodologies was evaluated for significance with a contingency chi-square test. Comparisons between the MIC values called by BMD testing versus those called by Etest were also assessed for significance by a Mann-Whitney-Wilcoxon *t* test.

Discrepancies between the method under evaluation and BMD testing were categorized as follows: VME, susceptible by the test method under evaluation and resistant by BMD testing (with values divided by total number of resistant isolates reported by the reference method); ME, resistant by the test method under evaluation and susceptible by BMD (with values divided by total number of susceptible isolates reported by the reference method); ME, a discrepancy between the test method under evaluation and reference methods involving an intermediate, SDD, or nonsusceptible result (with values divided by the total number of isolates). Pearson correlation coefficient calculations (*r* values) were calculated using GraphPad Prism to compare levels of MIC value agreement between the log2-adjusted MIC values determined by BMD testing and Etest for each isolate.

### Reproducibility study.

Initial analyses yielded BMD MICs at the higher range (1 to 4 mg/liter) for MRSA isolates. To confirm reproducibility of these higher-range MICs, the 20 isolates with the highest MICs were independently retested in the Center for Antimicrobial Resistance and Microbial Genomics (CARMiG) at the University of Texas Health Science Center, Houston, TX, USA, using an independent stock of CPT. In addition, since isolates frequently gave an MIC value of 1 mg/liter, which, according to EUCAST guidelines, lies in the “area of technical uncertainty,” we also tested 20 representative isolates in a fashion similar to that listed above. All these isolates exhibited a consistent CPT MIC of 1 mg/liter. Isolates with values of 1 mg/liter were categorized as “susceptible” for data analyses. All MICs were determined in three biological replicates (three separate inoculums) on panels prepared in-house, spanning a doubling dilution range of 0.032 mg/liter to 8 mg/liter.

## RESULTS

### Ceftaroline MIC distribution.

**FIG 1:**
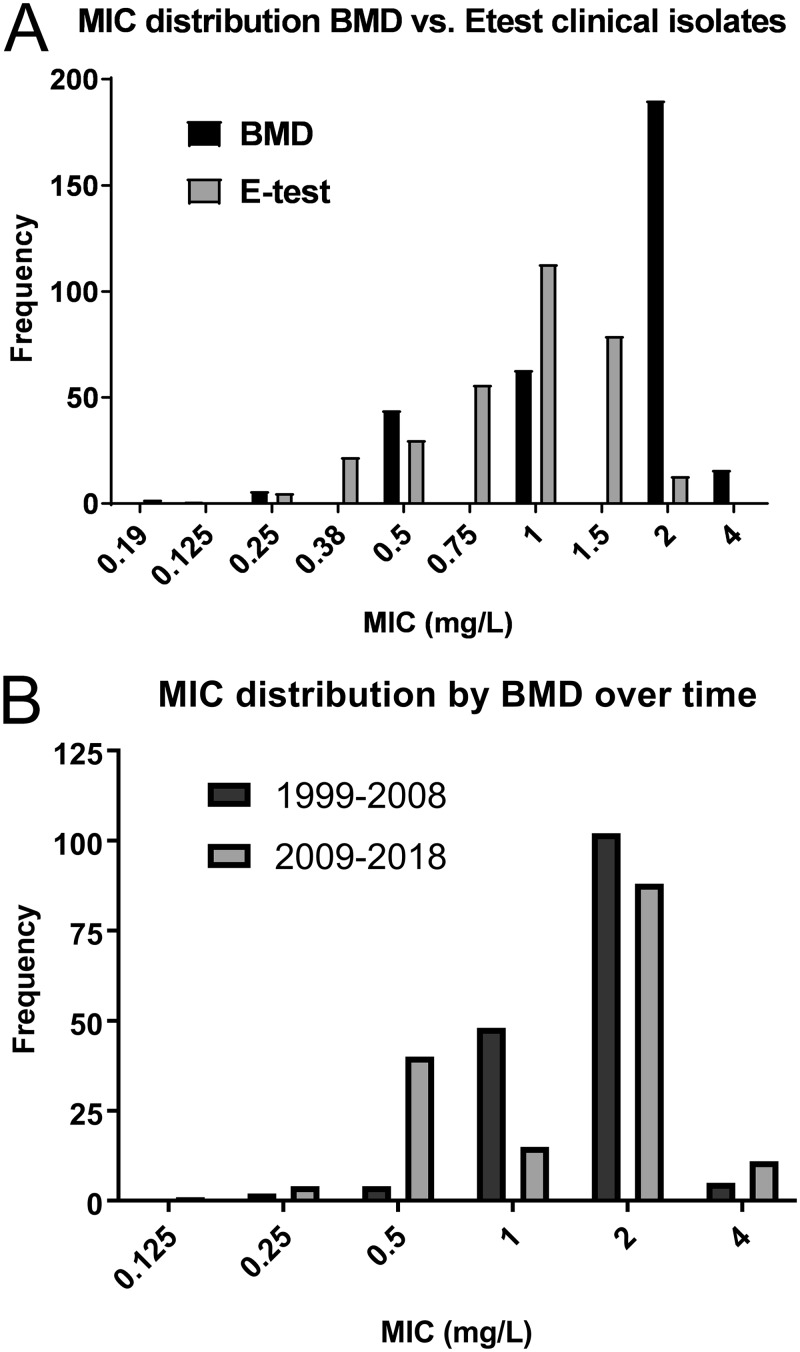
Ceftaroline (CPT) MIC distribution of clinical isolates. (A) Comparison of CPT susceptibilities of clinical isolates (*n* = 320) between broth microdilution (BMD) and Etest gradient strip methodologies. (B) MIC distributions of clinical isolates collected between 1999 and 2008 (*n* = 161) versus those collected between 2009 and 2018 (*n* = 159) by BMD.

**FIG 2:**
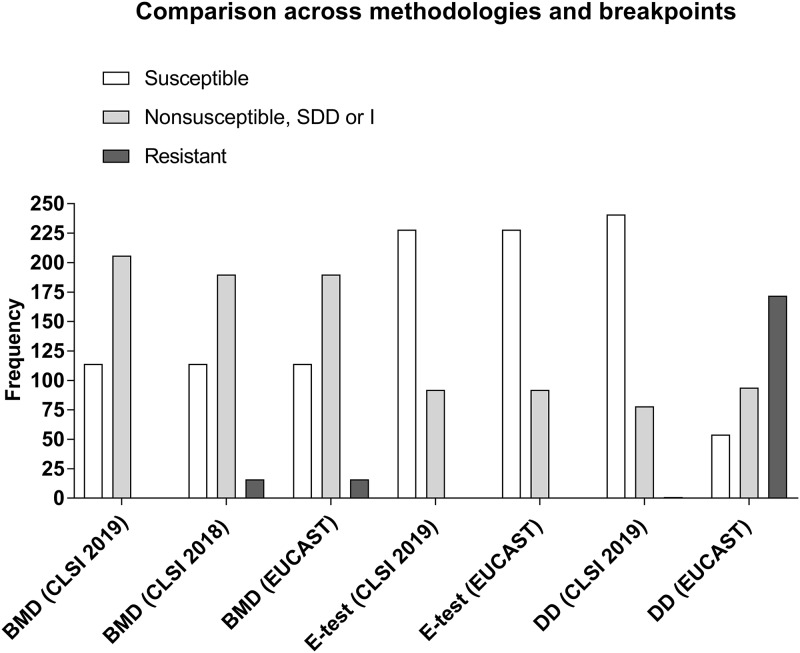
Frequency distribution of clinical isolates categorized as susceptible, nonsusceptible (including ATU), susceptible dose dependent (SDD)/intermediate (I), or resistant (R) across methodologies and agency guidelines. BMD, broth microdilution; DD, disk diffusion.

A total of 320 clinical MRSA isolates were collected from 9 hospitals across Santiago, Chile (1999 to 2018). The isolates were distributed in five time periods (3 years each) as follows: 1999 to 2002 (*n* = 93), 2003 to 2006 (*n* = 62), 2007 to 2010 (*n* = 45), 2011 to 2014 (*n* = 43), and 2015 to 2018 (*n* = 77). The sources of the clinical isolates were blood (*n* = 118), sterile fluids (*n* = 26), bone or tissue (*n* = 36), respiratory system (*n* = 78), skin (*n* = 55), and urine (*n* = 7). A total of 41 CA-MRSA isolates (see Materials and Methods for definition) were also assessed. BMD testing showed that CPT MIC distributions of the clinical isolates ranged from 0.125 mg/liter to 4 mg/liter, with MIC_50_ and MIC_90_ values of 2 mg/liter each (Fig. 1A), which is higher than the values previously reported for global MIC distributions of CPT in MRSA (MIC_50_ and MIC_90_ values of 0.5 mg/liter in the United States and 0.25 mg/liter in Europe) (16). In contrast, the MIC_50_ and MIC_90_ values determined for the 41 CA-MRSA isolates were significantly lower (*P* < 0.0001) at 0.5 mg/liter (range, 0.25 to 1 mg/liter; see Fig. S1 in the supplemental material). For reproducibility analyses, the 20 MRSA isolates with the highest MIC values and the 20 isolates with an MIC of 1 mg/liter, considered representative of an “ATU” according to EUCAST guidelines, were independently confirmed in triplicate by BMD testing. Among them, 39/40 gave identical MICs, whereas one isolate that previously exhibited an MIC of 2 mg/liter gave an MIC of 4 mg/liter in the independent laboratory. Of note, in each set of BMD measurements, the value determined for the quality control strain was confirmed to be within the range of 0.12 to 0.5 mg/liter. In fact, that strain consistently yielded an MIC of 0.25 mg/liter. Per the 2019 CLSI criteria, none of the isolates showed CPT resistance and 206/320 of the isolates fell in the SDD category (i.e., MIC values of 2 to 4 mg/liter). Per the EUCAST criteria, 16/320 isolates were CPT resistant and 190/320 were nonsusceptible (Table 1) (Fig. 2).

**TABLE 1:** Isolate categorization based on 2019 (revised) CLSI, 2018 CLSI, and 2018 EUCAST nonpneumonia guidelines

Isolate category	No. of isolates^*a*^							
	BMD (CLSI 2019)	BMD (CLSI 2018)	BMD (EUCAST)	Etest (CLSI 2019)	Etest (CLSI 2018)	Etest (EUCAST)	DD (CLSI)	DD (EUCAST)
Susceptible	114	114	114	228	228	228	241	54
Nonsusceptible			190			92		94
Susceptible dose dependent or intermediate^*b*^	206	190		92	92		78	
Resistant		16	16				1	172

a Data represent numbers of clinical isolates classified into the indicated susceptibility categories per broth microdilution (BMD), Etest, or gradient disk diffusion (DD) results.

b Susceptible dose-dependent, intermediate, and nonsusceptible data are grouped together for future error rate determinations.

In addition, we looked more closely at the CPT MIC distributions across time by comparing the isolates collected between 1999 and 2008 (*n* = 161) to those obtained between 2009 and 2018 (*n* = 159). The MIC_50_ and MIC_90_ values for the two sets were 2 mg/liter each (Fig. 1B), indicating that the high CPT MICs have been present for the past 2 decades. Furthermore, the isolates obtained from invasive infections (*n* = 179, i.e., the isolates collected from blood or other sterile sources) had similar MICs (MIC_50_ and MIC_90_ values of 2 mg/liter).

### MIC agreement between BMD and Etest.

We evaluated the performance of the Etest in relation to BMD testing (as the gold standard) (Fig. 2; see also Table S2 in the supplemental material). The MIC_50_ and MIC_90_ values according to Etest were 1 and 1.5 mg/liter, respectively, with the MIC distributions clearly showing that the Etest frequently underestimated the MIC values relative to the BMD values (Fig. 1A). EA was 82% (262/320, *P* < 0.0001), and the Pearson correlation coefficient value for the log2 adjusted binned MIC values for each isolate in the comparisons between BMD testing and Etest was low (*r* = 0.612).

Under the 2019 CLSI breakpoints, there was 51% (164/320, *P* < 0.0001) CA between BMD testing and Etest with no VME or ME and a 48% (154/320) ME rate (see Table 3). Of note, most of the ME were derived from isolates that fell in the SDD category by BMD but were catalogued as susceptible by Etest (42%, 134/320) (Table S6). Indeed, the majority of the lower values reported by Etest occurred with isolates in the 1 to 4 mg/liter CPT MIC range by BMD testing.

Using the EUCAST nonpneumonia guidelines, the CA of the results from Etest and BMD was also 51% (162/320, *P* < 0.0001). However, the VME rate was 81% (13/16) with no ME and a ME rate of 45% (144/320) (see Table 3). The ME was mostly attributable to the Etest underestimating the MIC and categorizing “nonsusceptible” strains as fully susceptible (38%, 121/320) (Table S6).

### Evaluation of the performance of the disk diffusion methodology.

Disk diffusion testing is still widely used for assessment of CPT susceptibility and is a more affordable alternative than automated testing or Etest. Thus, we sought to evaluate the performance of this method relative to BMD testing. The CA between disk diffusion (30 μg CPT) and BMD with 2019 CLSI criteria was 55% (177/320 [Table S3]; *P* < 0.0001). There was no VME with a ME and ME rates of 1% (1/320) and 44% (144/320), respectively (see Table 3). The latter mainly derived from the fact that the disk diffusion method underestimated the MIC, categorizing isolates as “nonsusceptible” compared to BMD (135/320, 42%) (Table S6).

CA between disk diffusion (5 μg CPT) and BMD with the nonpneumonia EUCAST guidelines was only 36% (115/320), with 6% (1/16) VME, 35% (40/114) ME, and 51% (164/320) ME (see Table 3) (*P* < 0.0001). These results were largely due to the disk diffusion method overestimating the MIC of isolates categorized as “nonsusceptible” by BMD (i.e., calling them resistant) (Fig. 2; see also Table 3 and Table S4).

### Comparison between EUCAST and CLSI breakpoints.

Due to the variation in CPT breakpoints across agencies, we sought to evaluate the level of agreement between EUCAST and CLSI (Fig. 2) (see Table 3). Comparison of BMD with the 2019 CLSI versus EUCAST nonpneumonia guidelines yielded a 95% CA (304/320) with only a 5% (16/320) ME (see Table 3; *P* < 0.05). For Etest comparisons between 2019 CLSI and EUCAST, the CA was 100%. Thus, the CLSI and EUCAST breakpoints performed similarly in comparisons of BMD testing and Etest results.

The EUCAST and CLSI guidelines are inherently variable for disk diffusion as they are based on different CPT disk concentrations (5 and 30 μg, respectively). The zone diameter cutoff values for resistance, however, are very similar (Table S1). Thus, comparisons of the EUCAST nonpneumonia and CLSI guidelines for disk diffusion performance were carried out using EUCAST as the baseline reference (since it utilizes disks with a lower concentration of CPT) (Table S5). The CA was 25% (115/320, *P* < 0.0001) with a high VME of 70% (120/172) and a ME of 38% (120/320) derived from the CLSI disk diffusion guidelines underestimating isolates deemed “resistant” by the EUCAST guidelines (see Table 3) (Fig. 2). Of note, using the CLSI disk diffusion guidelines as the reference, there was still a large discrepancy, with a high ME of 50% (120/241), no VME, and a ME of 38% (120/320) (see Table 3).

### The impact of revising CLSI breakpoints: a comparison between 2018 and 2019 criteria.

Since the CLSI guidelines for CPT were recently changed (Table S1), we evaluated the impact of this revision. Under the 2018 criteria, similarly to EUCAST, 5% of the isolates (16/320) were resistant by BMD testing. The revised 2019 criteria yielded no resistant isolates (Table 1) (Fig. 2). Comparing BMD testing and Etest with the old CLSI breakpoints yielded a CA similar to that seen with the 2019 criteria (51%, 164/320, *P* < 0.0001) but increased the VME to 81% (13/16) with no ME and a ME rate of 45% (143/320) (see Table 3). Most of the ME resulted from isolates considered susceptible by BMD but intermediate (MIC = 2 mg/liter) by Etest (38%, 120/320) (Table S6).

Comparing BMD versus disk diffusion with 2018 criteria yielded a CA of 53% (171/320, *P* < 0.0001), similar to results seen with the 2019 criteria. However, unlike the 2019 criteria, which yielded a VME of 0, the 2018 guidelines gave a VME of 63% (10/16), the same 1% ME (1/114), and a 43% ME (see Table 3). Thus, revision of the CLSI breakpoints has resulted in significant improvements in reducing serious discrepancies.

For both BMD testing and the Etest, the CA between the 2018 CLSI guidelines and the current EUCAST recommendations was 100%.

### Evaluation with EUCAST pneumonia guidelines.

EUCAST has a unique set of breakpoints for respiratory isolates with clinical indications of pneumonia (Table S1). We performed a secondary analysis evaluating our respiratory isolates under these guidelines and observed important differences. Under the pneumonia breakpoints, 64% (206/320) of the isolates were defined as resistant by BMD, 29% by Etest (92/320), and 83% (266/320) by disk diffusion, which are considerably higher than the levels observed with nonpneumonia criteria (5% by BMD, 0% by Etest, and 54% by disk diffusion) (Tables 1 and 2; see also Fig. S2).

**TABLE 2:** Isolate categorization based on EUCAST pneumonia guidelines for respiratory system-derived isolates

Isolate category	No. of isolates^*a*^		
	BMD (EUCAST)	Etest (EUCAST)	DD (EUCAST)
Susceptible	114	228	54
Resistant	206	92	266

a Data represent numbers of clinical isolates classified into the indicated susceptibility categories per broth microdilution (BMD), Etest, or gradient disk diffusion (DD) results.

Comparing BMD and Etest, the performance was similar to that seen with the nonpneumonia criteria (CA, 52%, 166/320, *P* < 0.0001) with a ME rate of 18% (20/320) but the VME rate was reduced to 65% (134/206) compared to the 81% rate obtained under nonpneumonia guidelines (Table S7). Evaluating BMD versus disk diffusion, the CA was 78% (250/320, *P* < 0.0001), yielding a VME rate of 2% (5/206), a higher ME of 40% (46/114), and a ME of 6% (19/320) (Table S7).

We also compared results across agencies. CA for BMD between 2019 CLSI and EUCAST under the pneumonia guidelines decreased to 36% (114/320, Table S7, *P* < 0.0001) relative to the 95% seen with the nonpneumonia criteria. The ME rate also increased to 64% (206/320) compared to the 5% seen with the nonpneumonia criteria. CA between 2019 CLSI and EUCAST for pneumonia for Etest was 71.3% (228/320, *P* < 0.0001) with no VME or ME and a ME rate of 29% (92/320).

CA between EUCAST pneumonia criteria and CLSI for disk diffusion using EUCAST (5 μg CPT) as a reference yielded a low CA of 23% (72/320, *P* < 0.0001), a high VME rate of 71% (188/266), and a ME rate of 39% (126/320) (Table S7). Using the CLSI disk diffusion guidelines as a reference, the discrepancies were still high with VME but with a high ME rate of 78% (188/241) and a ME rate of 39% (126/320). Thus, evaluation of respiratory isolates under the pneumonia guidelines increased the levels of the results with respect to the overall rates of resistance and contributed to an increase in the discrepancy rates since the breakpoints for resistance are lower (Table 2; see also Fig. S2).

## DISCUSSION

CPT is a relatively recently introduced broad-spectrum cephalosporin that possesses potent anti-MRSA activity. Previous studies have suggested that isolates belonging to some lineages of the ST5 genetic background seem to be inherently less susceptible to CPT than isolates belonging to other lineages, an observation that has been proposed to be related to clone-specific polymorphisms in PBP2a (18, 24). In order to further study this issue, we took advantage of a large collection of isolates obtained over a 20-year period in 9 hospitals (*n* = 320) in Santiago, Chile, a country where S. aureus infections are largely dominated by an ST5 MRSA clone. Previous data suggested that the global CPT MIC distributions for MRSA and methicillin-sensitive S. aureus (MSSA) were 0.25 to 1 mg/liter and 0.12 to 0.25 mg/liter, respectively (30). Our results indicate that the CPT MIC_50_ and MIC_90_ levels among our Chilean isolates are 4-fold to 8-fold higher than those reported from previous studies performed in other regions of the world (Fig. 1A). Despite variations in interpretation between CLSI and EUCAST breakpoints, the two agencies agree on a susceptibility breakpoint of 1 mg/liter for CPT based on the approved dosage regimen of 600 mg administered every 12 h (21, 22). Strikingly, 64% (206/320) of the MRSA isolates in our collection were CPT nonsusceptible (Table 1). The high CPT MICs observed (2 to 4 mg/liter) were highly reproducible, including in an independent laboratory. In contrast, the CA-MRSA isolates had lower MIC_50_ and MIC_90_ values (0.5 and 0.5, respectively; see Fig. S1 in the supplemental material) than the hospital-associated isolates, a finding that is consistent with previous reports (30). The high MIC distribution of the hospital-associated isolates was not associated with a temporal trend, since older isolates (obtained between 1999 and 2008) had values similar to those for the recent isolates (obtained between 2009 and 2018) (Fig. 1B). Furthermore, the high MIC values were not correlated with CPT usage in Chile, since it was not introduced until 2019. Thus, while the reported global rates of CPT resistance are still low (31), our findings suggest that lower susceptibility to CPT is an inherent characteristic of the ST5 Chilean-Cordobés lineage, which is the dominant MRSA clone in specific geographic regions of South America (8).

Accurate laboratory susceptibility testing is essential for proper usage of an antibiotic, a fact that has major clinical implications. Many diagnostic laboratories, particularly in North America and Europe, utilize automated systems for susceptibility determination, but the use of an Etest gradient strip has been widely adopted in clinical and research settings. Disk diffusion testing is used predominantly in developing countries due to its affordability and accessibility. Additionally, disk diffusion is used for susceptibility determinations of novel and recently approved agents for which automated systems and gradients strips are unavailable and have yet to be standardized (32).

We aimed to compare the levels of reliability and performance of the gradient Etest strip or disk diffusion against BMD (as the gold standard technique; Table 3). In general, there was good essential agreement between the MIC values determined by BMD testing and Etest (Table 3), though the Pearson correlation coefficient level of the MIC values across the spectrum was low. The MIC_50_ and MIC_90_ values, as determined using the Etest, were 1 and 1.5 mg/liter, respectively, suggesting that this methodology frequently underestimates MIC values relative to BMD testing (Fig. 1A) (23). BMD categorized 64% of our isolates as nonsusceptible, while the corresponding Etest value was lower at 29% (Table 1) (Fig. 2).

**TABLE 3:** Rates of agreement and error across methodologies (broth microdilution, Etest, and disk diffusion) evaluated under CLSI and EUCAST nonpneumonia guidelines^*a*^

Result	Rate (%)											
	BMD vs Etest	BMD vs Etest (CLSI 2019)	BMD vs Etest (CLSI 2018)	BMD vs Etest (EUCAST)	BMD (CLSI 2019) vs BMD (EUCAST)	BMD (CLSI 2018) vs BMD (EUCAST)	Etest (CLSI 2018 and 2019) vs Etest (EUCAST)	BMD (CLSI 2019) vs DD (CLSI)	BMD (CLSI 2018) vs DD (CLSI)	BMD vs DD (EUCAST)	DD (EUCAST) vs DD (CLSI)^*b*^	DD (CLSI) vs DD (EUCAST)^*c*^
Essential agreement	82											
Categorical agreement		51.3	51	51	95	100	100	55.3	53.4	36	25	25
Very major error (R by reference, S by test)		0	81.3	81.3	0			0	62.5	6.3	69.8	0
Major error (S by reference, R by test)		0	0	0	0			1	1	35.1	0	50
Total minor error (discrepancy in I or SDD classification)		48	45	45	5			44.4	43.1	51.3	37.5	37.5

a BMD, broth microdilution; DD, disk diffusion; S, susceptible; I, intermediate; SDD, susceptible dose dependent; R, resistant.

b EUCAST DD guidelines used as reference.

c CLSI DD guidelines used as reference.

Our study showed low (∼51%) CA in comparisons of BMD to the Etest across 2018 and revised 2019 CLSI breakpoints or across EUCAST breakpoints (Table 3). The EUCAST performance resembled that seen using the 2018 CLSI criteria. Notably, the transition to the new 2019 CLSI breakpoints successfully prevents the Etest from underestimating nonsusceptibility but increases the issue of MIC underestimation in isolates that fall in the SDD category relative to the gold standard. The level of agreement between CLSI and EUCAST across the BMD or Etest results is reassuringly high (Table 3).

The disk diffusion methodology applied to our isolates had low CA in comparison to the results seen with the gold standard across the 2019 CLSI, 2018 CLSI, and EUCAST nonpneumonia breakpoints (55%, 53%, and 36%, respectively; Table 3). The EUCAST criteria, in particular, had higher error rates than the 2019 CLSI criteria. Interestingly, the 2019 CLSI guidelines for disk diffusion testing categorized only 25% of our isolates as nonsusceptible, which is lower than that seen with the BMD or Etest determinations (Fig. 2) (Table 1). Under the EUCAST criteria, however, disk diffusion categorized 83% (266/320) of our isolates as nonsusceptible with particularly high rates of resistance (54%, 172/320) relative to those reported by BMD or Etest (Fig. 2) (Table 1). Thus, for disk diffusion, CLSI criteria underestimated nonsusceptibility (i.e., VME) while EUCAST overestimated it (i.e., ME). Since the CLSI and EUCAST guidelines are based on different CPT disk concentrations (30 and 5 μg, respectively), agreement between them is low and discrepancy levels are high (Table 3; see also Table S5). Thus, the different concentrations of CPT in the disks across the CLSI and EUCAST criteria directly impact the reliability of the disk diffusion methodology as a whole and support the arguments for better standardization of the disks or for complete revision of zone diameter breakpoints.

Reevaluation of respiratory isolates under the EUCAST pneumonia guidelines yielded overall rates of resistance determined with the BMD, Etest, and disk diffusion methods (64%, 29%, and 83%, respectively) (Table 2) that were higher than the nonpneumonia indications (Table 1; see also Fig. S2). There was also an impact on agreement and, in some cases, an increase in the levels of discrepancy in the comparisons of the performances of testing methodologies across the CLSI and EUCAST guidelines (Table S7). In a scenario in which there were relevant clinical indications to evaluate respiratory isolates under the pneumonia guidelines, the rates of CPT-resistant MRSA would be higher.

In conclusion, CPT nonsusceptibility has been dominant in Chile in the clinical setting over the last 20 years as determined on the basis of analysis of the isolates examined in our study. Detection of nonsusceptibility across methods is highly variable and inconsistent. While there is good agreement between the CLSI and EUCAST guidelines with the BMD and Etest methodologies, there is high discordance with the disk diffusion methodology. The Etest underestimates MICs relative to the gold standard. The EUCAST disk diffusion methodology, producing the lowest levels of agreement and the highest error rates, drastically overestimates resistance. The old CLSI guidelines from 2018 perform similarly to the EUCAST guidelines, but the recent revision in the CLSI guidelines has improved their reliability. We provide data that argue for better standardization of CPT breakpoints across agencies. We also show that Etest reliability could benefit from lowering the breakpoints specifically for Etest categorization. Finally, the highest level of discordance was seen with disk diffusion, arguing for revision of breakpoints across the CLSI and EUCAST guidelines or for standardization of the concentration of CPT in the disks. Thus, our results clearly show the inherent variability in antimicrobial susceptibility testing across testing methodologies, suggesting that interpretation of susceptibilities should be performed with caution and that clinical decisions need to be carefully weighed in particular scenarios. This approach is especially important if the MIC or the disk diffusion zone diameter reported falls on the edge of the susceptibility breakpoints set by regulatory agencies. Further standardization of techniques, breakpoints, and instruments will be necessary to improve agreement across agencies.

## Supplementary Material

Supplemental file 1
