# Supplementary material for: A Multicenter Study To Evaluate Ceftaroline Breakpoints: Performance in an Area with High Prevalence of Methicillin-Resistant Staphylococcus aureus Sequence Type 5 Lineage
Source: J Clin Microbiol. 2019 Aug 26;57(9):e00798-19. doi: 10.1128/JCM.00798-19 (PMC6711923; doi:10.1128/JCM.00798-19)
Supplement: Supplemental file 1 [file JCM.00798-19-s0001.pdf]

Supplementary Material

Supplementary Figures

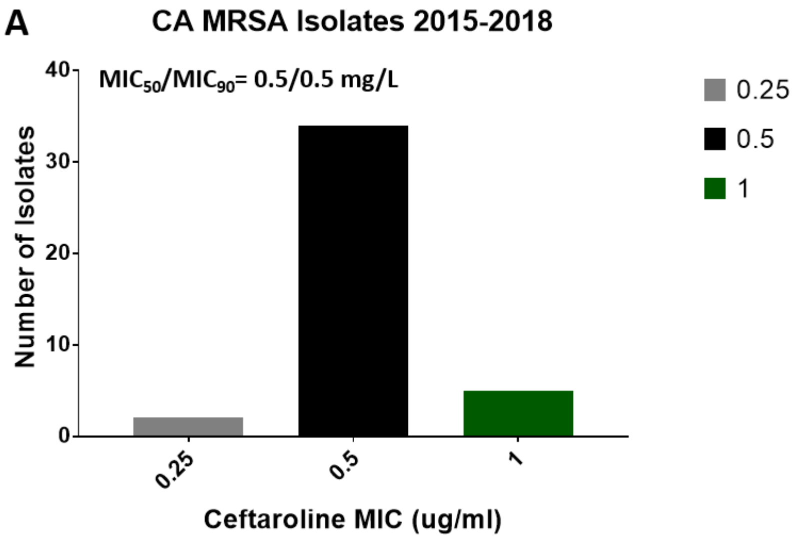

Figure S1: Minimum inhibitory concentration (MIC) distribution of community-associated MRSA (CA-MRSA) isolates (n=41) by broth microdilution

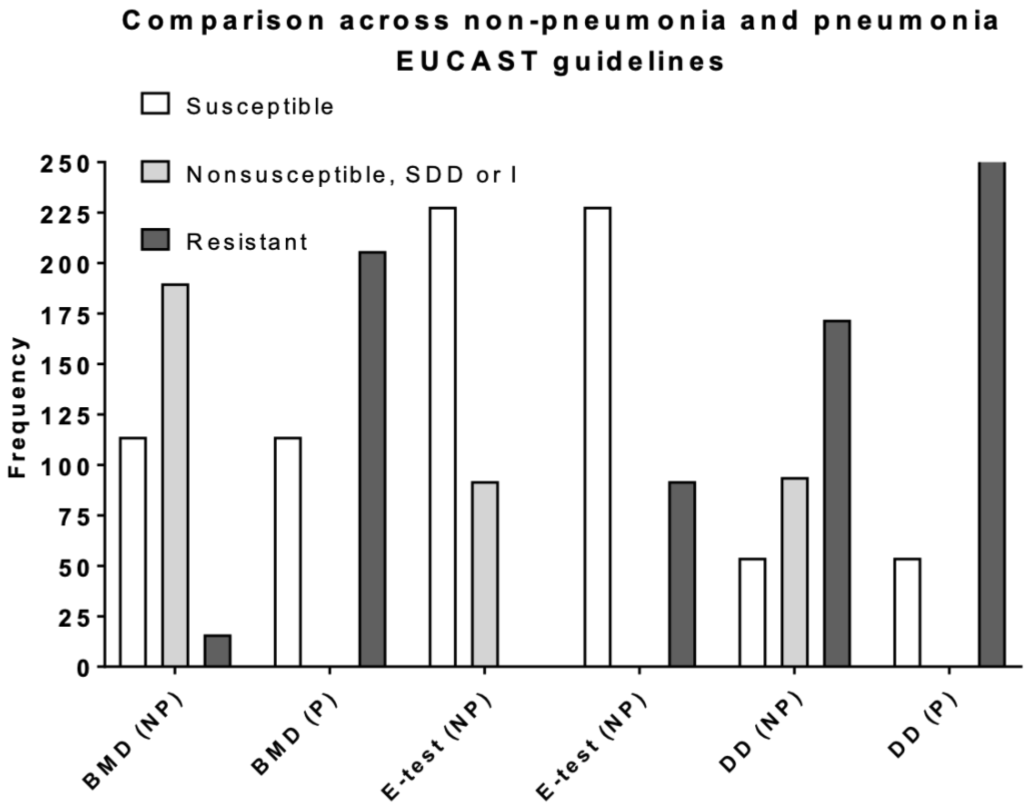

Figure S2: Frequency distribution of clinical isolates categorized as susceptible, nonsusceptibile (including ATU), susceptible dose-dependent (SDD)/ intermediate (I) or resistant (R) across methodologies when comparing non-pneumonia (NP) guidelines with pneumonia (P) guidelines. BMD: broth microdilution, DD: disk diffusion.

Supplementary Tables

	MIC (mg/L) CLSI 2019	MIC (mg/L) CLSI 2018	MIC (mg/L) EUCAST (non-pneumonia)	MIC (mg/L) EUCAST (pneumonia)	Zone diameter (mm) CLSI 2019 30ug CPT	Zone diameter (mm) EUCAST (non-pneumonia) 5 ug CPT	Zone diameter (mm) EUCAST (pneumonia) 5 ug CPT
Susceptible	≤ 1	≤ 1	≤ 1	≤ 1	≥ 25	≥ 20	≥ 20
Intermediate	-	2	-	-	-	-	
Susceptible dose-dependent	2-4		-	-	20-24	-	
Resistant	≥ 8	≥ 4	>2	>1	≤ 19	< 17	< 20
Area of technical uncertainty			1	1		19-20	19-20

Table S1: Minimum inhibitory concentration (MIC) and zone diameter breakpoints for ceftaroline (CPT) according to each agency.

		E-test strip								
	MIC values (mg/L)	0.19	0.25	0.38	0.5	0.75	1	1.5	2	Sum
Broth microdilution	0.125			1						1
	0.25	1	1	4						6
	0.5	1	4	15	17	3	4			44
	1			1	7	16	19	18	2	63
	2			1	5	34	81	59	10	190
	4				1	3	9	2	1	16
	Sum	2	4	22	30	56	113	79	13	

Table S2: Minimum inhibitory concentration (MIC) value distribution by broth microdilution versus E-test. Nonsusceptible MIC values as per CLSI and EUCAST breakpoints indicated in red.

Broth microdilution (mg/L)	Disk diffusion CLSI (mm)																		
	37	35	34	33	32	31	30	29	28	27	26	25	24	23	22	21	20	19	Sum
0.125										1									1
0.25		1	1				2	1	1										6
0.5			2	2	6	4	11	7	5	4	2	1							44
1	1			1		5	5	3	7	10	11	12	1	3	2	1		1	63
2	1				2	2	8	3	11	24	35	39	38	17	7	2	1		190
4							1	1	1	3	3	1	4	1	1				16
Sum	2	1	3	3	8	11	27	15	25	42	51	53	43	21	10	3	1	1	

Table S3: Agreement between inhibition zones (mm) determined by disk diffusion methodology using the CLSI ceftaroline disk (30 ug) versus minimum inhibitory concentration (MIC) value by broth. Nonsusceptible MIC or zone diameter values as per CLSI breakpoints indicated in red.

Broth microdilution (mg/L)	Disk diffusion EUCAST (mm)														
	32	25	24	23	22	21	20	19	18	17	16	15	14	6	Sum
0.125			1												1
0.25		2	2	1	1										6
0.5	1	2	3	6	6	11	10	2	2			1			44
1				1		1	1	5	6	10	15	22	2		63
2				1			3	5	27	28	63	49	13	1	190
4						1		4		5	3	3			16
Sum	1	4	6	9	7	13	14	16	35	43	81	75	15	1	

Table S4: Agreement between inhibition zones (mm) determined by disk diffusion methodology using the EUCAST ceftaroline disk (5 ug) versus minimum inhibitory concentration (MIC) value by broth. Nonsusceptible MIC or zone diameter values as per EUCAST breakpoints indicated in red.

Disk Diffusion EUCAST (mm) \ Disk diffusion CLSI (mm)	37	35	34	33	32	31	30	29	28	27	26	25	24	23	22	21	20	19	Sum
32									1										1
25		1					1	1	1										4
24					1		2		1	1	1								6
23			1			2	4			1				1					9
22			1	1	2		2		1										7
21			1	1	2	1	1	4	1	1		1							13
20					1	1	4	1	3	3	1								14
19						1	1	2	2	3	2	1	2	2					16
18	1						1	2	3	5	11	3	3	5	1				35
17	1						1	1	5	1	8	13	9	2	2				43
16					1	3	2	4	1	13	15	22	14	4	2				81
15				1	1	3	7		6	14	11	11	9	3	5	3	1		75
14							1				2	1	6	4				1	15
6												1							1
Sum	2	1	3	3	8	11	27	15	25	42	51	53	43	21	10	3	1	1	

Table S5: Agreement between inhibition zones (mm) determined by disk diffusion methodology using the CLSI ceftaroline disk (30 ug) versus the EUCAST disk (5ug). Nonsusceptible MIC or zone diameter values as per EUCAST or CLSI breakpoints indicated in red.

	BMD vs. E-test	BMD vs E-test (CLSI 2019)	BMD vs E-test (CLSI 2018)	BMD vs E-test (EUCAST)	BMD (CLSI 2019) vs BMD (EUCAST)	BMD (CLSI 2018) vs BMD (EUCAST)	E-test (CLSI 2018 and 2019) vs E-test (EUCAST)	BMD (CLSI 2019l) vs DD (CLSI)	BMD (CLSI 2018) vs DD (CLSI)	BMD vs DD (EUCAST)	DD (EUCAST) vs DD (CLSI 2019)*	DD (CLSI) vs DD (EUCAST) **
Total Minor Error (Discrepency in "I" or "SDD" classification)		48%	45%	45%	5%	-	-	44.4%	43.1%	51.3%	37.5%	37.5%
Minor Error 1 (SDD or I by reference, S by test)		41.8%	6.3%	37.8%	0	-	-	42.2%	39.1%	1.3%	21.3%	0.3%
Minor Error 2 (S by reference, SDD or I by test)		6.3%	37.5%	6.3%	0	-	-	2.2%	2.2%	7.8%	0.3%	21.3%
Minor Error 3 (R by reference, SDD or I by test)		0	1%	1%	0	-	-	0	1.9%	2.8%	16%	0
Minor Error 4 (SDD or I by reference, R by test)		0	0	0	5%	-	-	0	0	39.4%	0	16%

Table S6. Stratified minor error rates across methodologies (broth microdilution (BMD), E-test, disk diffusion (DD)) evaluated under CLSI and EUCAST non-pneumonia guidelines. S: susceptible, I: intermediate, SDD: susceptible dose-dependent, R: resistant. * EUCAST DD guidelines used as reference. ** CLSI DD guidelines used as reference.

	BMD vs E-test (EUCAST)	BMD (CLSI 2019) vs BMD (EUCAST)	BMD (CLSI 2018) vs BMD (EUCAST)	E-test (CLSI 2019 and 2018) vs E-test (EUCAST)	BMD vs DD (EUCAST)	DD (EUCAST) vs DD (CLSI)*	DD (CLSI) vs DD (EUCAST) **
Categorical Agreement	52%	35.6%	40.6%	71.3%	78%	22.5%	22.5%
Very Major Error (R by reference, S by test)	65%	0	0	0	2.4%	70.7%	0
Major Error (S by reference, R by test)	17.5%	0	0	0	40.4%	0	78%
Total Minor Error (Discrepency in "I" or "SDD" classification)	0	64.4%	59%	29%	6%	39.4%	39.4%
Minor Error 1 (SDD or I by reference, S by test)	0	0	0	0	0	15%	0.3%
Minor Error 2 (S by reference, SDD or I by test)	0	0	0	0	6%	0.3%	15%
Minor Error 3 (R by reference, SDD or I by test)	0	0	0	29%	0	24%	0
Minor Error 4 (SDD or I by reference, R by test)	0	64.4%	59%	59.4%	0	0	24%

Table S7: Rates of agreement and error across methodologies (broth microdilution (BMD), E-test, disk diffusion (DD)) evaluated under CLSI and EUCAST pneumonia guidelines applied to respiratory derived isolates. S: susceptible, I: intermediate, SDD: susceptible dose-dependent, R: resistant. * EUCAST DD guidelines used as reference. ** CLSI DD guidelines used as reference.
